# Postoperative Outcomes of Double-Barreled Wet Colostomy Versus Ileal Conduit After Pelvic Exenteration: A Systematic Review and Meta-analysis

**DOI:** 10.7759/cureus.98070

**Published:** 2025-11-29

**Authors:** Luis Alves, Bernardo F Pompeu, Vinnicius Duarte, Rubens Rigo, Gabriel Leal Barone, Lucas Guedes, Fernanda Formiga, Leonardo Borges

**Affiliations:** 1 General and Colorectal Surgery, Heliópolis Hospital, São Paulo, BRA; 2 General and Colorectal Surgery, University of São Caetano do Sul, São Paulo, BRA; 3 Medicine, University of São Caetano do Sul, São Paulo, BRA; 4 Colorectal Surgery, Santa Casa de São Paulo, São Paulo, BRA; 5 Colorectal Surgery, Heliópolis Hospital, São Paulo, BRA; 6 Urology, Albert Einstein Hospital, São Paulo, BRA

**Keywords:** ileal conduit, pelvic exenteration, systematic review and meta-analysis, urinary diversion, wet colostomy

## Abstract

Ileal conduit (IC) remains the standard urinary diversion after pelvic exenteration, but double-barreled wet colostomy (DBWC) has regained interest as a viable alternative when simultaneous fecal diversion is required, as it allows the diversion of both urinary and digestive tracts through a single stoma, thereby simplifying postoperative management and reducing the overall functional burden on patients. To compare postoperative complications and operative outcomes of these two procedures, a systematic search was conducted across PubMed, Scopus, and the Cochrane Central Register of Controlled Trials for studies published up to March 2025. Pooled odds ratios (ORs) and mean differences (MDs), along with 95% confidence intervals (CIs), were calculated using random-effects models, with heterogeneity assessed by the I² statistic, and analyses performed with R software, Version 4.4.1 (R Foundation for Statistical Computing, Vienna, Austria, https://www.R-project.org/). This review ultimately included five retrospective observational studies with 345 patients, of whom 195 (56.5%) underwent DBWC and 150 (43.4%) underwent IC. The analysis found no significant differences between DBWC and IC for several key outcomes, including urinary leak (OR 0.38; 95% CI 0.13-1.05; p = 0.06), pyelonephritis (OR 0.43; 95% CI 0.18-1.08; p = 0.07), uretero-enteric stenosis (OR 0.99; 95% CI 0.21-4.61; p = 0.98), severe complications (Clavien-Dindo ≥ III) (OR 1.54; 95% CI 0.70-3.39; p = 0.28), electrolyte disturbances (OR 0.74; 95% CI 0.27-2.02; p = 0.55), hospital stay (MD -4.46 days; 95% CI -12.77 to 3.84; p = 0.29), operative time (MD 6.28 minutes; 95% CI -157.74 to 170.29; p = 0.94), and mortality (OR 0.55; 95% CI 0.18-1.70; p = 0.30). Notably, sensitivity analysis revealed that DBWC was associated with a significantly lower risk of intestinal leak after the exclusion of one influential study (OR 0.21; 95% CI 0.07-0.67; I² = 0%). In conclusion, the findings suggest that DBWC is a safe and feasible alternative to IC for urinary reconstruction after pelvic exenteration, with comparable postoperative outcomes.

## Introduction and background

Colorectal and gynecological cancers are common malignancies that frequently infiltrate adjacent pelvic structures. In colorectal cancer, invasion of surrounding organs occurs in 20%-40% of locally advanced cases, with bladder involvement reported in approximately 3%-17% of patients [1-6]. In gynecological malignancies, particularly cervical cancer, adjacent organ invasion is observed in about 15% of advanced or recurrent cases [7,8]. For urinary diversion after pelvic exenteration, the Bricker ileal conduit (IC) remains the standard approach [9]. However, when both urinary and fecal diversions are necessary, a single-stoma technique, known as wet colostomy, has been proposed as an alternative [10].

The double-barreled wet colostomy (DBWC), first described by Carter in 1989, was designed for patients requiring both fecal and urinary diversions [10]. It uses a 15-cm segment of sigmoid colon fashioned into a urinary conduit alongside an end colostomy, both exteriorized through a single stoma. This single-site approach aims to lessen the burden of managing two stomas, reducing overall costs and improving patient well-being, but remains debated due to concerns about urinary infections, odor, and metabolic complications [11]. Managing two distinct stomas may increase the complexity of postoperative self-care, doubling the risk of peristomal skin complications and higher rates of leakage or irritation. This dual-stoma configuration also demands higher long-term costs associated with materials and nursing support.

A previous meta-analysis included only three observational studies, but more recent publications have expanded the evidence base. This highlights the need for an updated and comprehensive assessment. Therefore, the objective of this systematic review and meta-analysis is to compare postoperative outcomes of DBWC and IC after pelvic exenteration.

## Review

Methods

This systematic review followed the Preferred Reporting Items for Systematic Reviews and Meta-Analysis (PRISMA) guidelines [12]. The study protocol was registered in the International Prospective Register of Systematic Reviews (PROSPERO) with registration number CRD420251011497 [13]. As this study is based on a systematic review and meta-analysis of previously published data, it is exempt from ethical clearance.

Search Strategy

A systematic search was performed across PubMed, Scopus, and Cochrane Central Register of Clinical Trials for studies published up to March 2025. The search strategy was as follows: ("pelvic exenteration" OR "pelvic exenterations" OR "Cystectomy" OR "Radical Cystectomy" OR "pelvic exenterat" OR ureterostomy) AND ("wet colostomy" OR "double-barrelled" OR "uro-colostomy" OR “Neobladder” OR "double-barreled wet colostomy" OR “DBWC”) AND (“Urostomy” OR "ileal conduit" OR "Bricker" OR "Bricker conduit" OR "ileal urinary diversion" OR “SUD”).

Eligibility Criteria

We included observational studies that compared DBWC with IC in adult patients undergoing pelvic exenteration. The exclusion criteria were (1) other urinary reconstructions like ureterostomy or neobladder; (2) studies without a control group; (3) publications deemed ineligible for inclusion, such as single-arm studies, case reports, conference abstracts, meta-analyses, reviews, and animal studies; (4) studies with overlapping patient populations; and (5) procedures not performed in the elective setting.

Data Extraction and Endpoints

Two authors (L.E.B.M.A. and V.N.T.D.) independently selected articles that met the inclusion criteria, using the Rayyan app (Rayyan Systems Inc., Cambridge, MA, USA), and extracted data from the selected studies. After that, the extracted data were placed in an Excel spreadsheet (Microsoft Corp., Redmond, WA, USA). Any disagreements were resolved by consensus or, if necessary, by consulting a third author (B.F.P.). The outcomes assessed were (1) pyelonephritis, (2) urinary leak, (3) intestinal leak, (4) Clavien-Dindo score, (5) operative time, (6) length of hospital stay, (7) mortality, (8) electrolyte abnormalities, and (9) uretero-enteric stenosis.

Quality Assessment

Two authors (L.E.B.M.A. and V.N.T.D.) independently evaluated the quality of the included studies using the Revised Cochrane Risk-of-Bias tool for non-randomized studies of interventions (ROBINS-I) [14]. Each study was categorized as presenting low, moderate, serious, critical risk of bias, or no information across seven domains: (1) confounding, (2) selection of participants, (3) classification of interventions, (4) deviations from intended interventions, (5) missing data, (6) measurement of outcomes, and (7) selection of the reported result. Disagreements were resolved by consensus through consultation with the senior author (B.F.P.).

Statistical Analysis

We pooled odds ratios (OR) for binary outcomes and mean differences (MD) for continuous endpoints, with 95% confidence intervals (CI). A random-effects model was used for all outcomes. Statistical significance was defined as p < 0.05. Heterogeneity was assessed using the Cochran Q test and I² statistics, with p-values lower than 0.10 and I² > 25% being considered significant for heterogeneity. For outcomes with substantial heterogeneity, we used Baujat plots to assess each study’s contribution to the overall effect and heterogeneity. Furthermore, we also performed leave-one-out sensitivity analyses by systematically removing each study from the pooled estimates to ensure the robustness of the results. R Software, Version 4.4.1 (R Foundation for Statistical Computing, Vienna, Austria, https://www.R-project.org/), was used for statistical analysis. Additionally, the methodological quality and risk of bias of non-randomized studies were evaluated using the ROBINS-I tool.

Results

Study Selection and Characteristics

**Figure 1:**
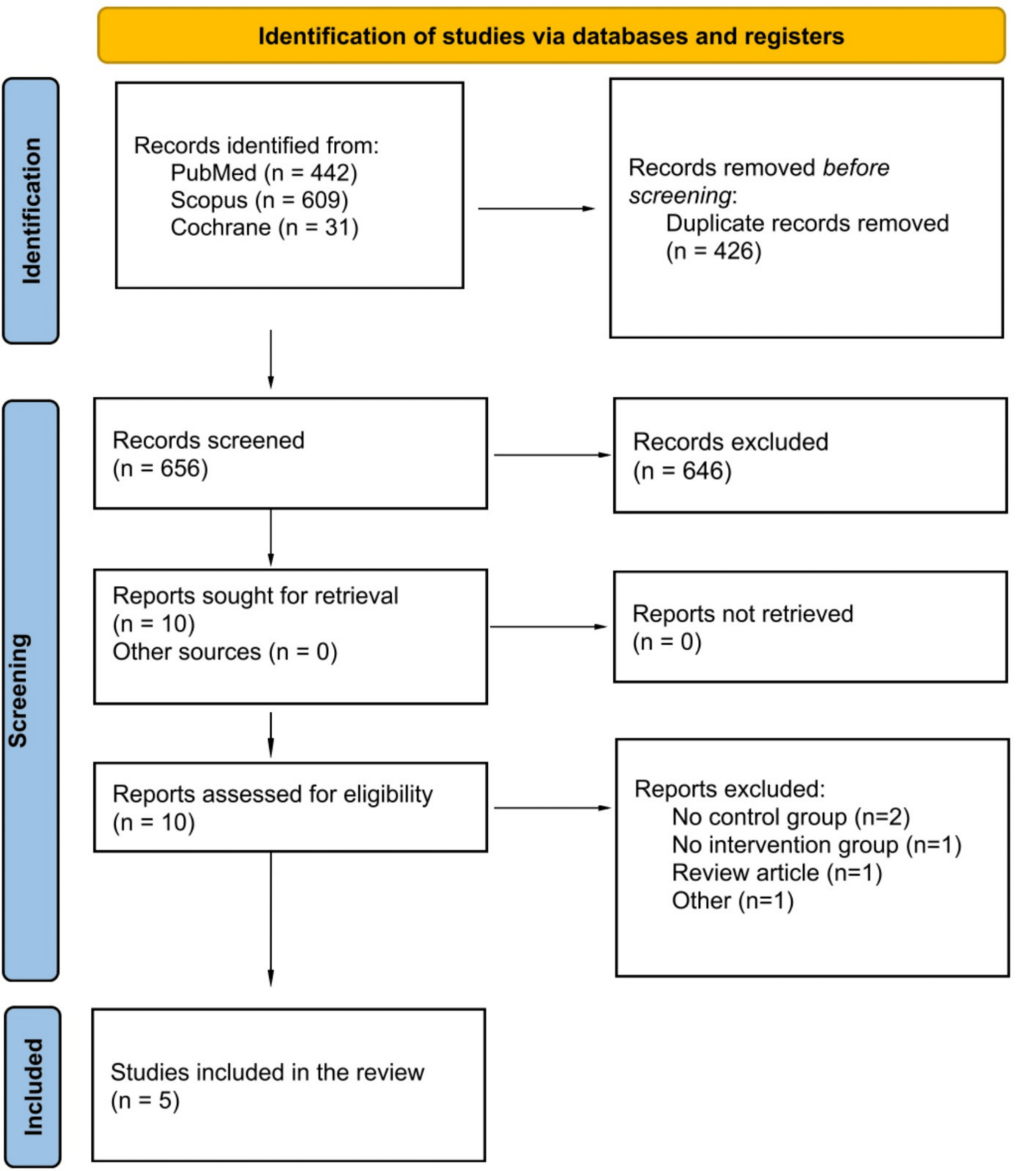
PRISMA flow diagram of the study screening and selection process

As shown in Figure 1, the initial search identified 1,082 records. After excluding 426 duplicates and 646 titles and abstracts, 10 studies were reviewed in full text. Of these, five met the eligibility criteria and were included in the meta-analysis.

In total, 345 patients underwent urinary diversion, of whom 303 (87.8%) had the procedure following pelvic exenteration. Among them, 195 (56.5%) underwent DBWC, while 150 (43.4%) received an IC [11,15-18]. Females accounted for 69.5% of the population [11,15-18]. The mean age was 55.6 ± 11.4 years in the DBWC group and 57.4 ± 12.2 years in the IC group. The mean body mass index (BMI) was 27.6 ± 5.6 kg/m² and 28.7 ± 6.3 kg/m², respectively [11,15-18].

Total pelvic exenteration, defined as the removal of all pelvic organs and classified as anterior (genitourinary tract) or posterior (rectal tract) using the uterus as reference, was reported in three studies with the following rates: 100% in Chokshi, 71.7% in Nguyen, and 65% in Lago [15-17]. Less extensive resections were also described, including anterior exenteration (Nguyen 17.9%; Lago 20%), posterior exenteration (Lago 15%), and laterally extended endopelvic resection (LEER) (Backes 26%; Lago 8%) [15,16,18]. Selected cases included pelvic bone involvement and lateral lymph node dissection [16]. Regarding oncological indications, gynecologic cancers predominated: 100% in Lago, 93.9% in Backes, 32% in Pavlov, 26.3% in Nguyen, and 11.3% in Chokshi. Colorectal cancer was also common, especially in Chokshi (67.9%) and Nguyen (63.1%), with lower proportions in Pavlov (42.5%) and Backes (3%) [11,16-18].

In the Pavlov cohort, recurrent disease and palliative intent accounted for 15.5% of IC cases and 43.2% of DBWC cases, primarily due to radiation-induced injury, highlighting a higher palliative rate in the DBWC group [11]. Preoperative radiotherapy was frequently used, either as neoadjuvant therapy for gynecologic cancers or as part of total neoadjuvant therapy (TNT) for colorectal cancer, with rates ranging from 60% to 100% [16-18]. Intraoperative radiotherapy, however, was less common and reported only in two studies [17-18]. Additional study characteristics are detailed in Tables 1-2.

**Table 1:** Baseline characteristics of the included studies R-Obs: retrospective observational study, NA: not available, BMI: body mass index, DBWC: double-barreled wet colostomy, IC: ileal conduit.

Author	Country	Design	Sample number		Female n (%)		Age (years), mean ± SD		BMI (kg/m^2^), mean ± SD		Follow-up (months), mean ± SD	
			DBWC	IC	DBWC	IC	DBWC	IC	DBWC	IC	DBWC	IC
Chokshi et al. (2011) [17]	USA	R-Obs	43	10	27 (62.7)	5 (50)	56.4 ± 9.4	62.4 ± 10.1	NA	NA	NA	NA
Backes et al. (2013) [18]	USA	R-Obs	12	11	12 (100)	11 (100)	55.1 ± 9.1	55.0 ± 15.0	29.1 ± 5.8	26.6 ± 7.0	43.7 ± 37.7	
Pavlov et al. (2014) [11]	Serbia	R-Obs	104	77	77 (74.0)	42 (54.5)	54.0 ± 10.7	54.8 ± 11.2	NA	NA	NA	NA
Nguyen et al. (2023) [16]	Australia	R-Obs	16	23	3 (18.7)	14 (60.8)	56.6 ± 18.0	66.5 ± 11.5	26.5 ± 5.3	29.7 ± 5.7	17.5 ± 7.1	50.8 ± 43.0
Lago et al. (2023) [15]	Spain	R-Obs	20	29	20 (100)	29 (100)	62.0 ± 11.0	57.0 ± 11.0	NA	NA	NA	NA

**Table 2:** Distribution of tumor types in the included studies DBWC: double-barreled wet colostomy, IC: ileal conduit, CRC: colorectal cancer, GC: gynecologic cancer, UC: urologic cancer, SCCA: squamous cell carcinoma of the anus, LMS: leiomyosarcoma, SM: suspected malignancy, R: recurrent disease, RT: radiation therapy.

Author	Indication of urinary diversion (n, %)		Pathology (n, %)			Previous RT (n, %)		Intraoperative RT (n, %)	
	DBWC	IC	DBWC		IC	DBWC	IC	DBWC	IC
Chokshi et al. [17]	TPE: 43 (100)	10 (100)	CRC: 32 (74.4)		4 (40)	33 (76.7)	6 (60.0)	18 (41.8)	4 (40.0)
			GC: 5 (11.6)		1 (10)				
			UC: 2 (4.6)		3 (30)				
			SCCA: 2 (4.6)		0 (0)				
			LMS: 2 (4.6)		1 (10)				
			SM: 0 (0)		1 (10)				
Backes et al. [18]	TPE: 12 (100)	11 (100)	NA		NA	12 (100)	11 (100)	6 (50.0)	NA
Pavlov et al. [11]	PE: 75 (72)	64 (83)	GC (R): 16 (15.3)		42 (50.6)	45 (43.2)	12 (15.5)	NA	NA
	Palliative: 12 (12)	10 (10)	GC: 0 (0)		3 (3.8)				
	Sequelae RT: 17 (16)	3 (4)	CRC: 20 (19.2)		18 (23.3)				
			CRC (R) colon: 3 (2.8)		6 (7.7)				
			CRC (R) rectal: 21 (20.1)		9 (11.6)				
			UC (R): 4 (3.8)		0 (0)				
Nguyen et al. [16]	Total PE: 16 (100)	12 (52.1)	CRC: 13 (81.2)		13 (81.2)	15 (93.7)	15 (65.2)	NA	NA
	Modified total PE: 0 (0)	2 (8.7)	GC: 2 (12.5)		8 (34.7)				
	Anterior PE: 0 (0)	7 (30.4)	UC: 0 (0)		1 (4.3)				
	Other: 0 (0)	2 (8.6)	Benign: 1 (6.2)		3 (13.0)				
Lago et al. [15]	Total PE: 40 (81.6)		GC: 49 (100)			NA	NA	NA	NA
	Anterior PE: 12 (24.4)								
	Posterior PE: 9 (18.3)								

Pooled Analyses of All Studies

**Figure 2:**
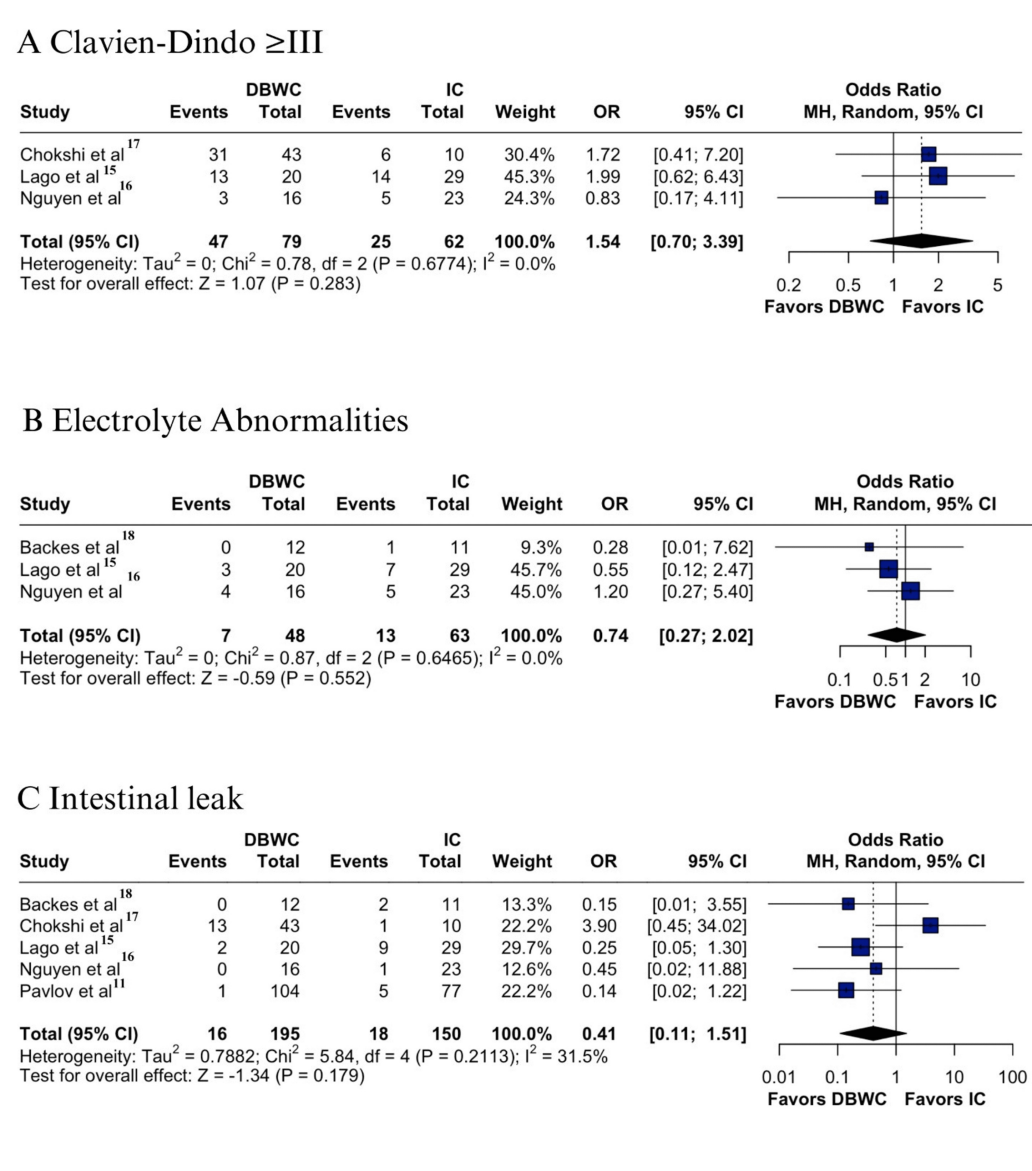
Forest plots of DBWC vs IC urinary diversion after pelvic exenteration: (A) Clavien-Dindo ≥ III, (B) electrolyte abnormalities, (C) intestinal leak

In the pooled analysis of patients undergoing pelvic exenteration with DBWC versus IC urinary diversion, no significant differences were observed in major complications or Clavien-Dindo grade ≥ III rates (59.4% vs 40.3%; OR 1.54; 95% CI 0.70-3.39; p = 0.283; I² = 0%) (Figure 2A) [15-17], nor in electrolyte disturbances (14.5% vs 20.6%; OR 0.74; 95% CI 0.27-2.02; p = 0.552; I² = 0%) (Figure 2B) [15,16,18]. Both outcomes demonstrated low heterogeneity. Similarly, intestinal leakage showed no significant difference between groups (8.2% vs 12.0%; OR 0.41; 95% CI 0.11 to 1.51; p = 0.179; I² = 31.5%) (Figure 2C) [11,15-18], with moderate heterogeneity.

**Figure 3:**
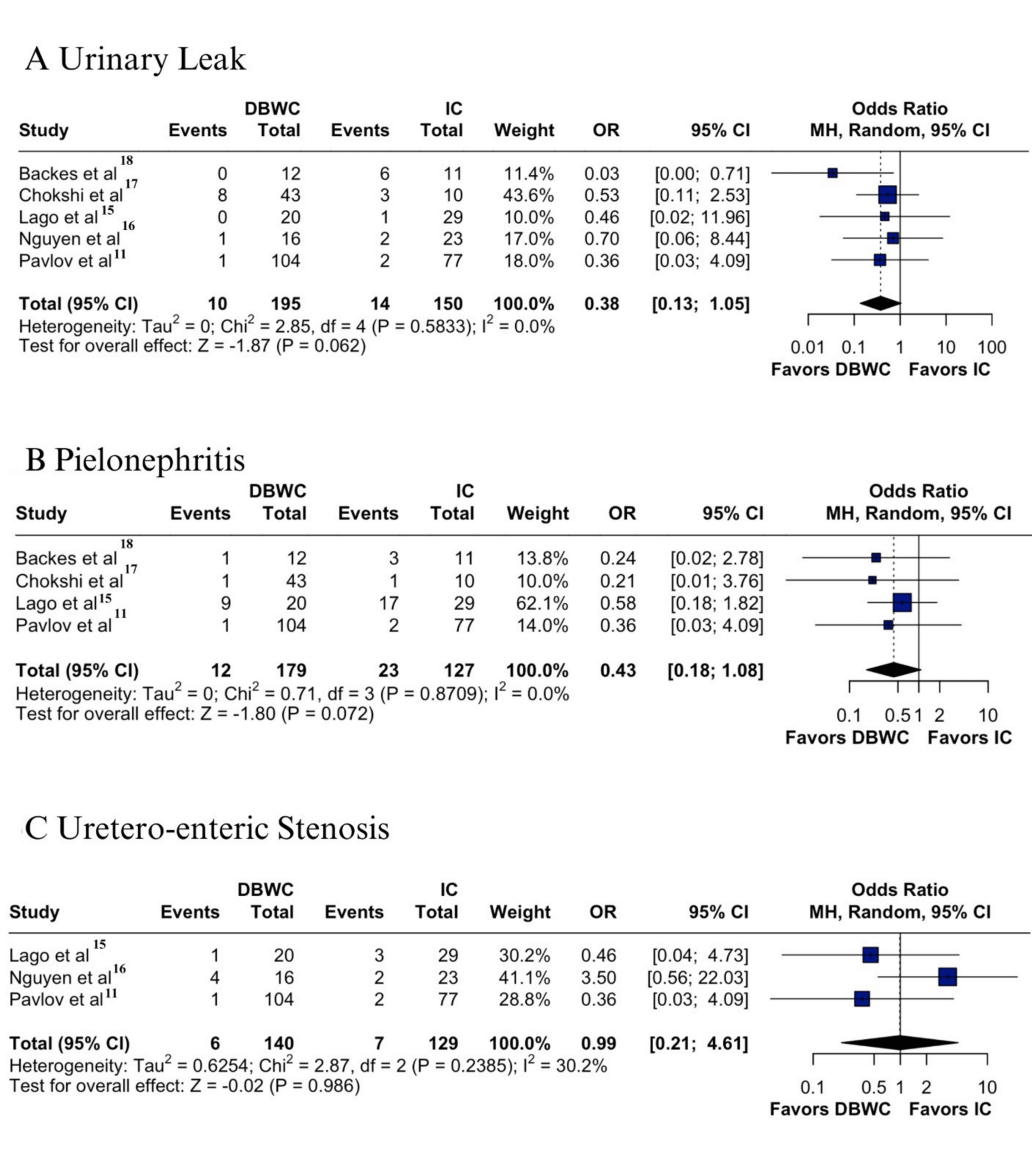
Forest plots of DBWC vs IC urinary diversion after pelvic exenteration: (A) urinary leak, (B) pyelonephritis, (C) uretero-enteric stenosis

Regarding urinary complications, no statistically significant differences were observed for urinary leak (5.1% vs 9.3%; OR 0.38; 95% CI 0.13 -1.05; p = 0.062; I² = 0% (Figure 3A) [11,15-18], pyelonephritis (6.7% vs 18.1%; OR 0.43; 95% CI 0.18-1.08; p = 0.072; I² = 0%) (Figure 3B) [11,15,17,18], or uretero-enteric stenosis (4.2% vs 5.4%; OR 0.99; 95% CI 0.21- 4.61; p = 0.98; I² = 30.2%) (Figure 3C) [11,15,16]. Notably, only uretero-enteric stenosis presented moderate heterogeneity. 

**Figure 4:**
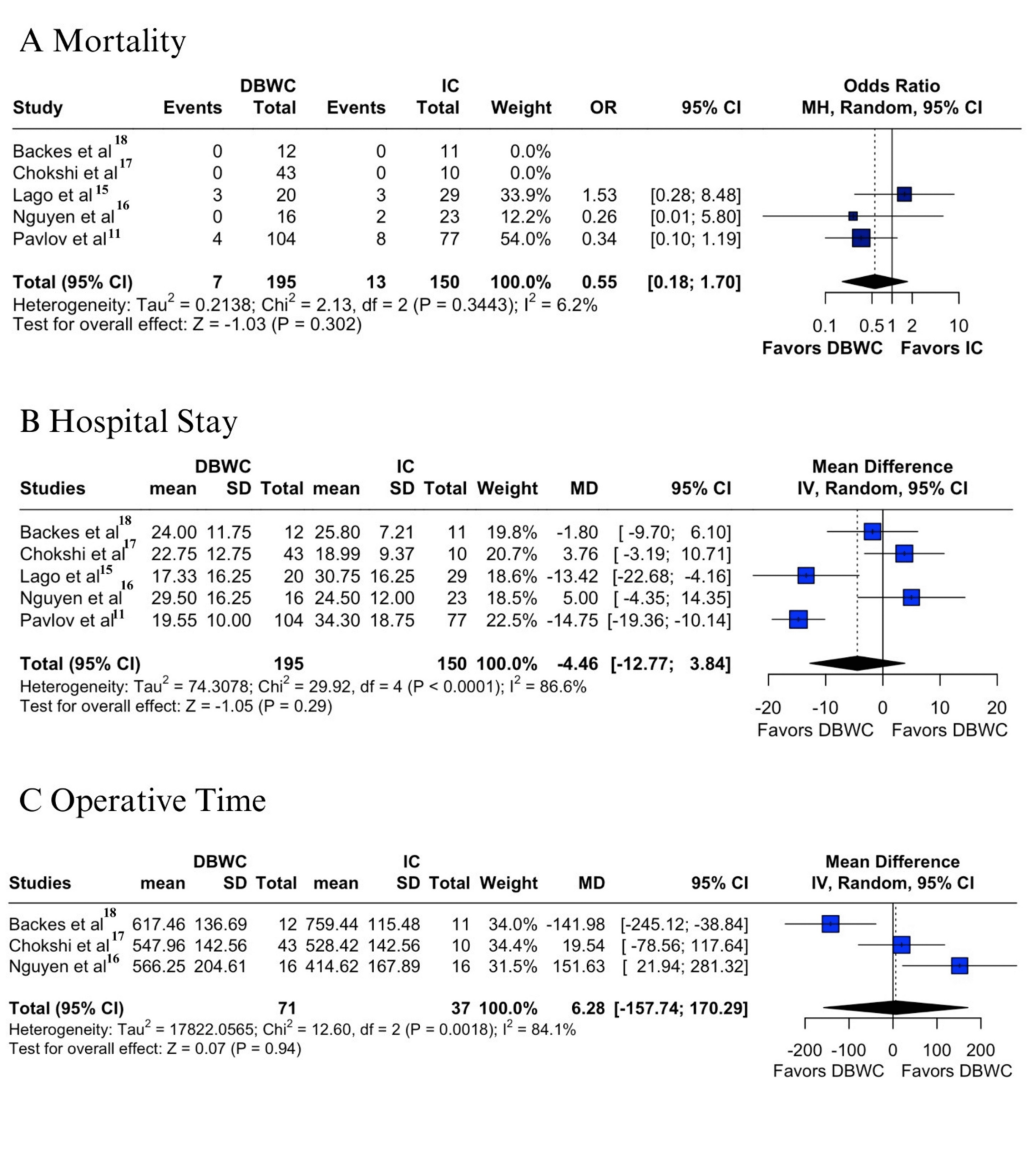
Forest plots of DBWC vs IC urinary diversion after pelvic exenteration: (A) mortality, (B) hospital stay, (C) operative time

Mortality rates were also comparable, with a trend toward lower values in the DBWC group (3.5% vs 8.6%; OR 0.55; 95% CI 0.18-1.70; p = 0.302; I² = 6.2%) (Figure 4A) [11,15,17,18], but without statistical significance. Finally, hospital stay did not differ significantly between groups (MD -4.46 days; 95% CI -12.77 to 3.84; p = 0.29; I² = 86.6%) (Figure 4B) [11,15-18], and operative time showed high heterogeneity and similar results (MD 6.28 minutes; 95% CI -157.74 to 170.29; p = 0.94; I² = 84.1%) (Figure 4C) [16-18].

Sensitivity Analyses

**Figure 5:**
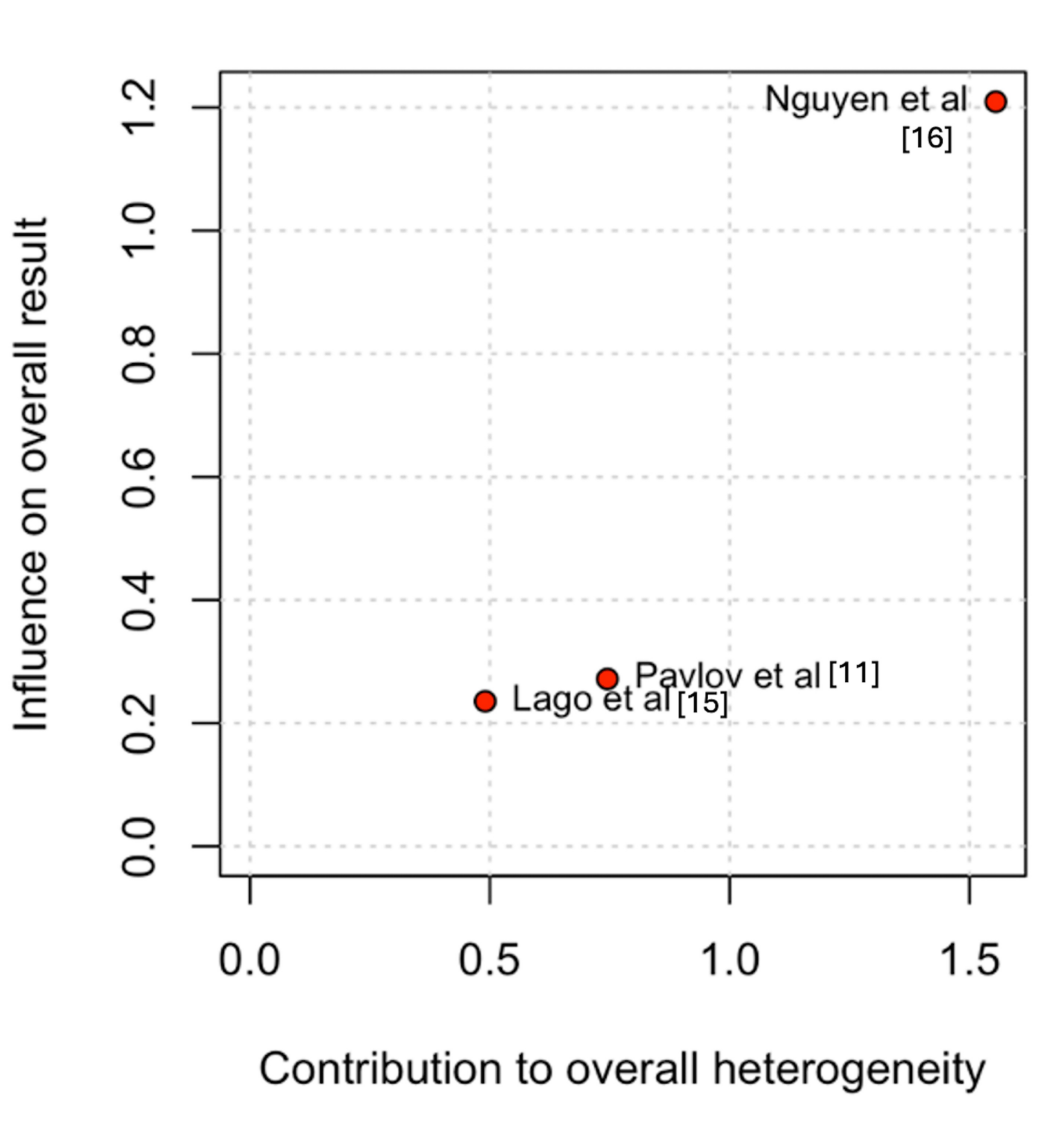
Baujat plot for uretero-enteric stenosis

**Figure 6:**
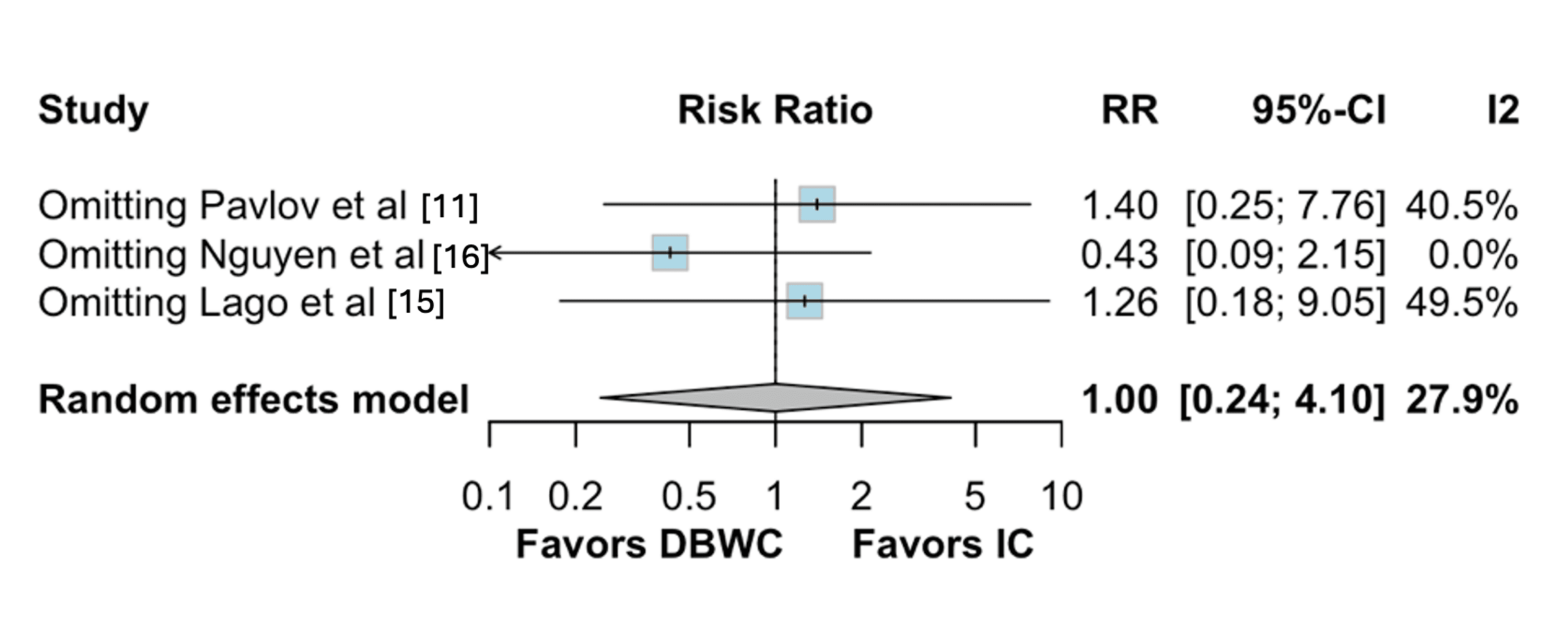
Leave-one-out sensitivity analysis for uretero-enteric stenosis

The Baujat plot analysis identified the studies that contributed most to heterogeneity. For ureter-enteric stenosis, Nguyen et al. [16] emerged as the main contributor. However, exclusion of this study in the leave-one-out analysis did not alter the overall results (Figures 5-6) [11,15,16].

**Figure 7:**
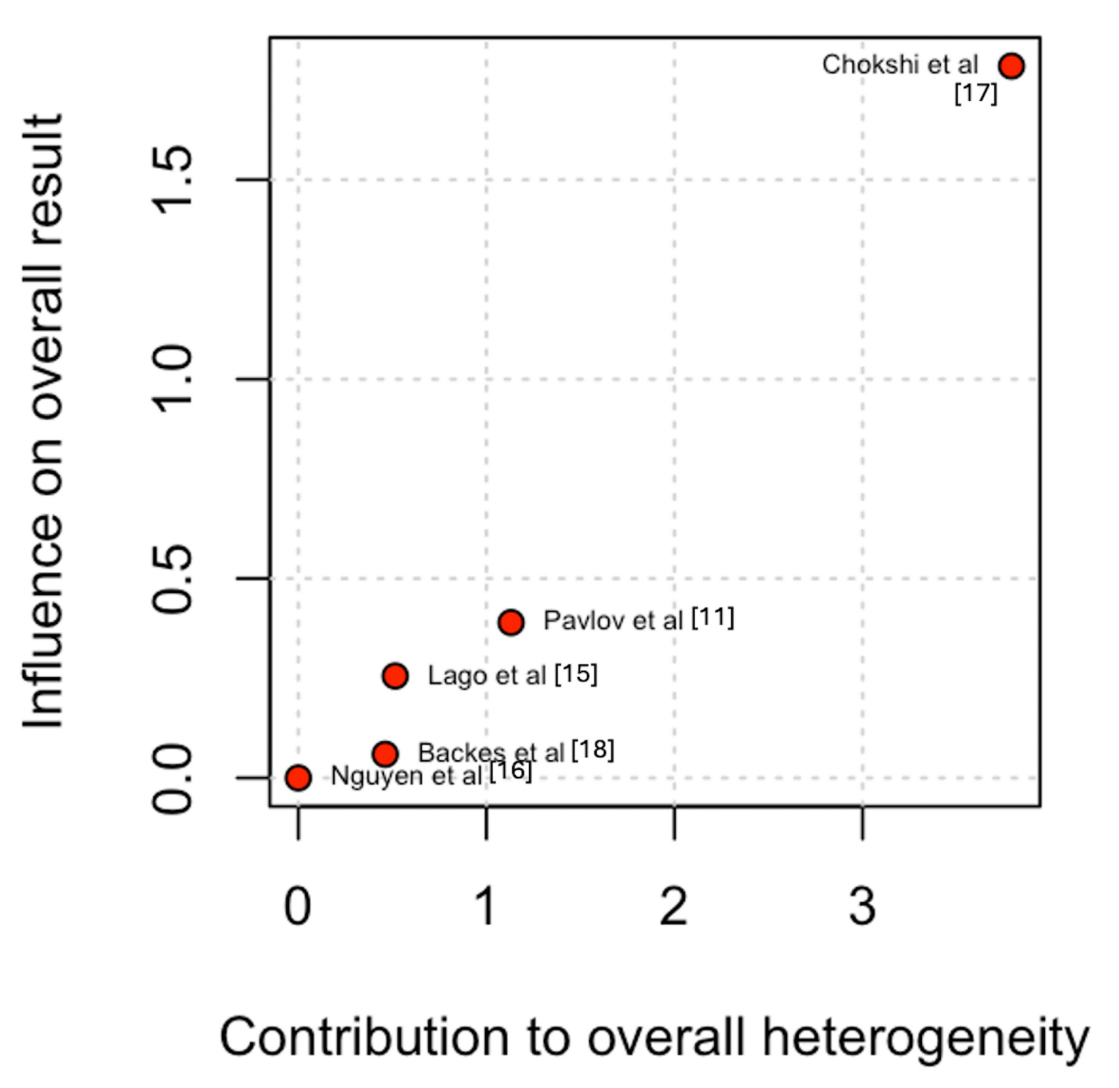
Baujat plot for intestinal leak

**Figure 8:**
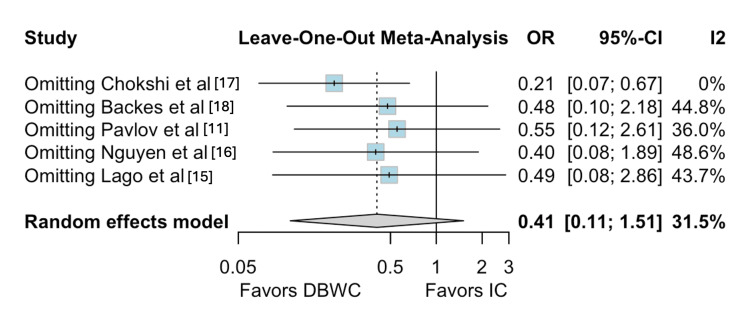
Leave-one-out sensitivity analysis for intestinal leak

For intestinal leak, Chokshi et al. [17] contributed the most to the observed heterogeneity. Leave-one-out sensitivity analysis demonstrated a statistically significant decrease in the incidence of intestinal leak in the DBWC group (OR 0.21; 95% CI 0.07 to 0.67; I² = 0%) (Figures 7-8) [11,15-18].

**Figure 9:**
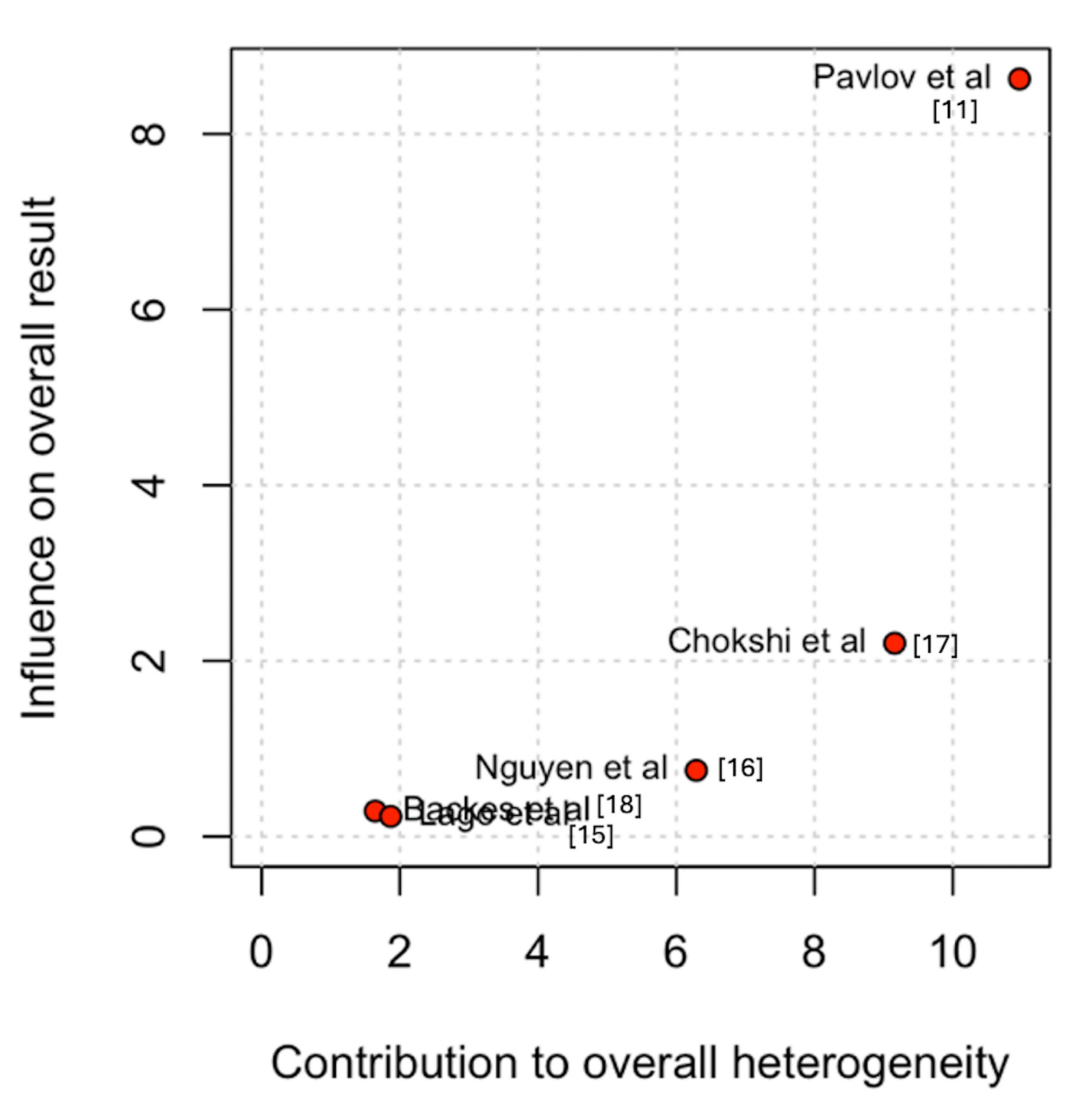
Baujat plot for hospital stay

**Figure 10:**
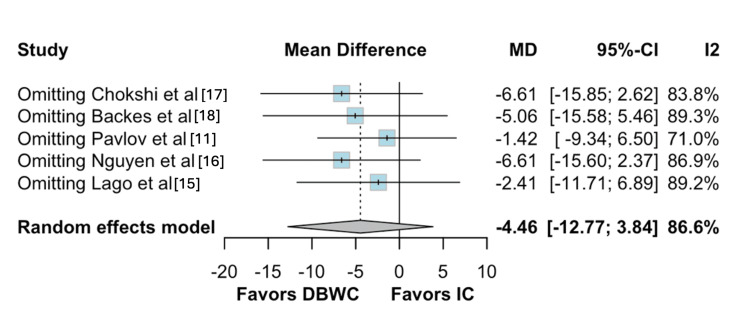
Leave-one-out sensitivity analysis for length of stay

Regarding hospital stay, Pavlov et al. [11] and Chokshi et al. [17] were the primary sources of heterogeneity. Nonetheless, the pooled estimate remained consistent after exclusion of these studies (Figures 9-10) [11,15-18].

**Figure 11:**
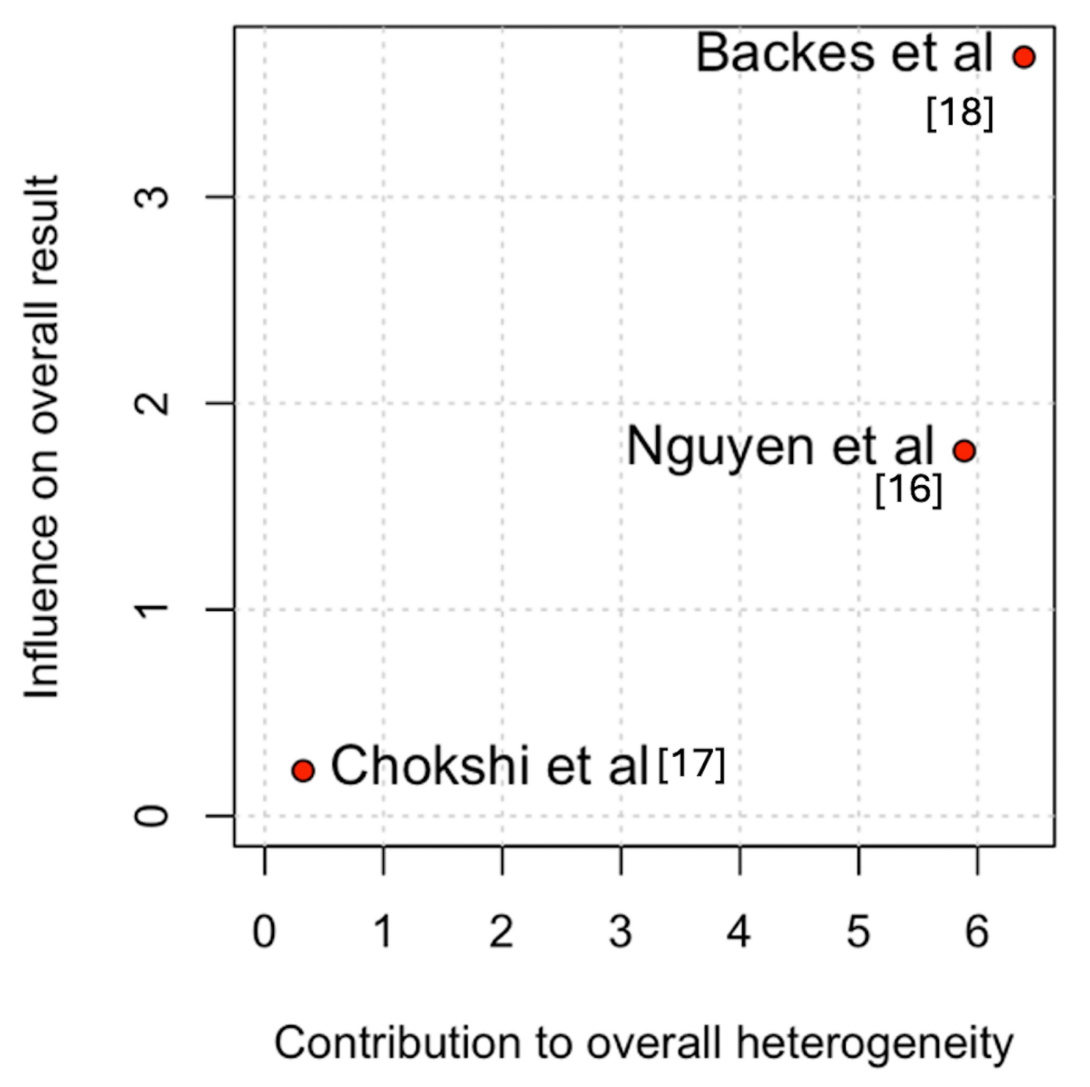
Baujat plot for operative time

**Figure 12:**
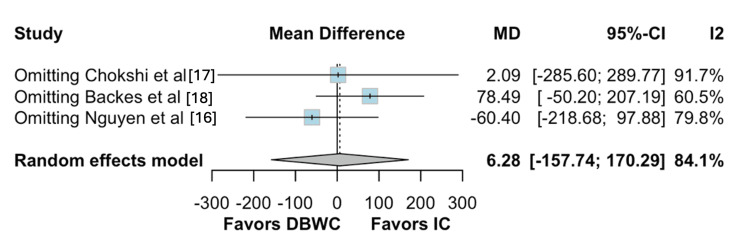
Leave-one-out sensitivity analysis for operative time

For operative time, Backes et al. [18] accounted for most of the heterogeneity (Figures 11-12) [16-18]. The leave-one-out analysis confirmed that removal of this study did not significantly affect the overall findings.

Quality Assessment

**Figure 13:**
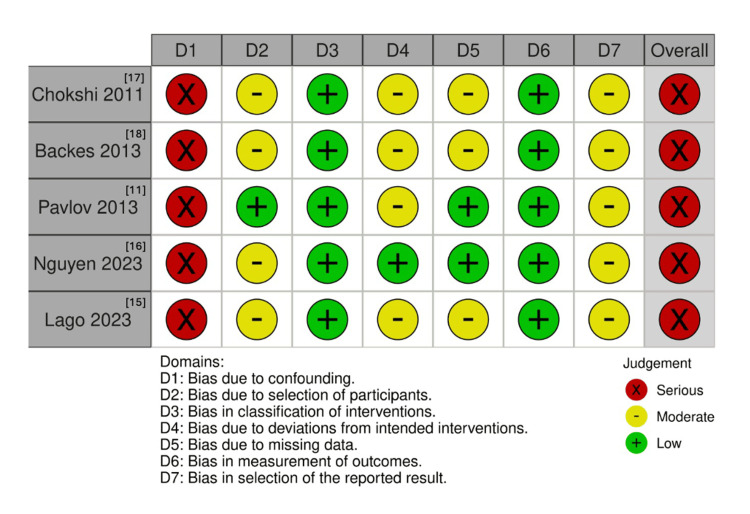
Critical appraisal of studies according to the Cochrane Collaboration’s tool for assessing risk of bias (ROBINS-I)

The individual risk-of-bias assessment using the ROBINS-I tool is illustrated in Figure 13.

All included studies were judged to have a serious risk of bias due to confounding, primarily because none applied randomization, matching, or statistical adjustment for prognostic factors [11,15-18]. Allocation to groups was generally based on institutional protocols or temporal factors, reducing comparability. Selection bias was considered moderate in most studies, except for Pavlov et al. [11], which included consecutive patients. Intervention classification was consistently low risk, while deviations from intended interventions were moderate in four studies due to variability in surgical practices [11,15-18]. Most studies had a moderate risk of bias due to missing data, except for two studies that were affected by unblinded retrospective assessments [11,16]. Measurement of outcomes was generally low risk, and all studies lacked protocol registration, leading to a moderate risk of selective reporting [11,15-18]. Overall, all studies were rated as serious risk of bias, given the consistent presence of serious confounding. Therefore, the current evidence should be interpreted with caution.

Discussion

In this systematic review and meta-analysis, five observational studies involving 345 patients undergoing urinary diversion were included. No statistically significant differences were observed between DBWC and IC regarding urinary fistula, pyelonephritis, uretero-enteric stenosis, Clavien-Dindo complications, electrolyte imbalances, operative time, length of hospitalization, or mortality. However, in sensitivity analysis, DBWC was associated with lower rates of intestinal leak (OR 0.21; 95% CI 0.07-0.67; I² = 0%).

DBWC has been considered a viable alternative for urinary reconstruction in patients undergoing pelvic exenteration. Although there is concern that using a colonic segment could increase the risk of urinary tract infections, available studies suggest that rates of infectious complications such as pyelonephritis are comparable or even lower when compared to IC [19-26]. Previous data on DBWC, particularly when performed with antireflux uretero-colonic anastomosis, have demonstrated satisfactory reservoir function and low infection rates. Nonetheless, from a urological perspective, the double-barreled configuration poses practical challenges, since combining fecal and urinary output in the same stoma often results in difficult hygiene maintenance, with continuous contamination by stool and urine [22,26].

Intestinal leak remains an important complication after pelvic exenteration. Webb and Symmonds described an incidence of 12.6%, particularly higher among patients with previous radiation exposure [19]. Fuccio et al. also highlighted potential predisposing factors such as prior abdominal surgery, diabetes, inflammatory bowel disease, smoking, low BMI, and connective tissue disorders [20]. In our analysis, although irradiated patients were more frequent in the DBWC group, this cohort showed a lower leak rate (8.2%). Notably, sensitivity testing indicated a lower incidence of intestinal leak in the DBWC group once a single influential study was excluded, suggesting that study-level characteristics may have influenced this outcome.

Urinary leak, though relatively infrequent, remains a relevant postoperative complication due to its potential to cause chemical peritonitis and sepsis. Reported rates following DBWC range from 0% to 14% [10,21-23]. Such leaks typically occur at the site of the uretero-colonic anastomosis [22]. Osorio Gullón et al. described only one case of urinary fistula after DBWC, successfully managed conservatively [22]. Across studies, the preferred management has been non-surgical, including percutaneous nephrostomy and radiologic drainage, with surgical revision rarely required. Pavlov et al. and Backes et al. reported complete resolution of fistulas with nephrostomy alone, while Nguyen et al. and Chokshi et al. achieved favorable outcomes using interventional radiology or drainage within 30 days [11,16-18]. These findings align with the review by Gan et al., who emphasized that most urinary fistulas after DBWC can be managed conservatively, with low morbidity and satisfactory resolution rates [23].

Beyond leak-related outcomes, overall morbidity has also been reported. Three studies in our review reported postoperative complications according to the Clavien-Dindo classification, allowing standardized comparison between DBWC and IC [15-17]. Although the DBWC group appeared to have a higher proportion of severe complications, interpretation is limited because events were reported cumulatively, without clarifying whether multiple complications occurred in the same patient [15-17]. Beyond the pooled analysis, individual series have reported widely variable morbidity rates after DBWC. Lopes de Queiroz et al. observed a 56% complication rate following pelvic exenteration [24], whereas Takada et al. reported no complications (0%) [21]. Osorio Gullón et al. [22] reported 15%, and Golda et al. [25] described rates as high as 78%. Long-term follow-up data for IC also demonstrate substantial morbidity: Madersbacher et al. reported 192 conduit-related complications in 87 of 131 patients, with a median follow-up of 98 months [26]. Collectively, these findings highlight the heterogeneity of outcomes across techniques and reinforce the importance of careful patient selection and individualized perioperative management.

This study has several limitations. All included studies were retrospective and non-randomized, making them inherently prone to bias. The heterogeneity in surgical approaches and oncologic indications further complicates direct comparisons between techniques. In particular, differences in the extent of surgery (total vs anterior pelvic exenteration) and patient profiles across studies may have influenced outcomes, potentially favoring less extensive procedures and limiting the generalizability of the findings. Moreover, the lack of long-term data on functional outcomes and quality of life is a significant limitation, especially given the high morbidity associated with pelvic exenteration. Another important constraint is the limited number of studies available on this topic. Additionally, the serious risk of bias due to confounding identified by the ROBINS-I assessment may have either over- or underestimated the observed effects, underscoring the need for cautious interpretation of the findings. These factors highlight the need for future prospective and randomized trials with larger cohorts to provide more reliable and generalizable evidence. Overall, these findings reinforce the importance of individualized decision-making when selecting urinary and fecal diversion strategies after pelvic exenteration. Although DBWC may offer potential advantages, the current evidence remains limited and heterogeneous. Future research should focus on prospective designs with standardized reporting of complications and long-term assessment of functional outcomes and quality of life. Such studies are essential to clarify which patient populations derive the greatest benefit from each reconstructive approach and to strengthen the evidence base guiding surgical decision-making.

## Conclusions

In this systematic review and meta-analysis, five observational studies including 345 patients undergoing urinary diversion were analyzed, of whom 303 (87.8%) had the procedure after pelvic exenteration. No statistically significant differences were found between DBWC and IC regarding urinary fistula, pyelonephritis, uretero-enteric stenosis, Clavien-Dindo complications, electrolyte disturbances, operative time, length of hospitalization, or mortality. However, sensitivity analysis suggested a potentially lower risk of intestinal leak in the DBWC group, highlighting the need for cautious interpretation. Further prospective studies with standardized methodologies are warranted to clarify long-term safety, functional outcomes, and quality-of-life implications. From a clinical perspective, DBWC may be particularly advantageous in scenarios where simultaneous fecal and urinary diversion is required, such as in patients undergoing extensive multivisceral resections or those with pre-existing bowel dysfunction that already necessitates a colostomy. In these contexts, consolidating both diversions into a single stoma may reduce the overall stoma burden, simplify postoperative care, and potentially improve patient adaptation. Conversely, in patients with favorable anatomy, good baseline functional status, or when a separate fecal diversion is not anticipated, IC remains an appropriate and widely established option.
